# Repetitive transcranial magnetic stimulation reduces nicotine dependence and potentially modulates white matter microstructure in smokers: a pilot study by diffusion spectrum imaging

**DOI:** 10.3389/fneur.2025.1653926

**Published:** 2025-10-17

**Authors:** Dongyan Chen, Zhiqiang Li, Mei Xie, Tao Wang, Ruiyang Li, Yao Chen, Siyin Li, Qiaoli Zhang, Yuting Ling, Xiaoyun Liang, Huan Mao, Lihao Zhai, Jianjun Zhang

**Affiliations:** ^1^Department of Radiology, Zhejiang Hospital, Hangzhou, China; ^2^Institute of Research and Clinical Innovations, Neusoft Medical Systems Co., Ltd., Shanghai, China; ^3^Yiruide Medical Equipment New Technology Co., Ltd., Wuhan, China

**Keywords:** repetitive transcranial magnetic stimulation, nicotine, smoking cessation, white matter, diffusion spectrum imaging

## Abstract

**Introduction:**

The present study aims to investigate the effects of repetitive transcranial magnetic stimulation (rTMS) on smoking cessation and white matter (WM) structure related to the mesolimbic dopamine pathway using diffusion spectrum imaging (DSI).

**Methods:**

The rTMS over the left dorsolateral prefrontal cortex (DLPFC) was repeated 10 times in 18 smokers. Quantitative anisotropy (QA) and fractional anisotropy (FA) were calculated for the anisotropy assessment, and mean diffusivity (MD), radial diffusivity (RD) and axial diffusivity (AD) were determined for the diffusivity evaluation. Nicotine dependence, and craving and withdrawal symptoms were evaluated using the Fagerström Test for Nicotine Dependence (FTND), the short version of the Tobacco Craving Questionnaire (sTCQ), the visual analogue scale (VAS), and the Minnesota Nicotine Withdrawal Scale (MNWS).

**Results:**

After 10 times of rTMS, the FTND, MNWS and VAS scores significantly decreased, when compared to baseline, and withdrawal symptoms were partially alleviated. Furthermore, cigarette consumption was significantly decreased by rTMS, and four participants completely stopped smoking after rTMS treatment. Importantly, the smokers only had a reduction of AD in the right nucleus accumbens (NAc) fibers after rTMS, and a strong positive correlation was observed between the change in cigarette consumption and change in AD values in the right NAc fibers after rTMS treatment.

**Discussion:**

These results suggest that rTMS over the left DLPFC is a potential effective strategy for nicotine dependence and craving, which is probably due to the modulation of the right NAc fibers. The right NAc emerged as a region of interest that warrants further investigation as a potential therapeutic target.

## 1 Introduction

Smoking has been recognized as one of the most widespread and enduring forms of addiction, with its well-documented adverse health effects (1). China is presently the largest producer and consumer of tobacco in the world, and the number of tobacco-related deaths in China is proposed to increase from approximately one million in 2010 to two million annually by 2030 and three million by 2050, unless widespread smoking cessation occurs (2).

The nature of tobacco addiction primarily stems from the effects of nicotine on the central nervous system (CNS) (3). Similar to other addictive substances, nicotine activates the mesolimbic dopamine pathway which originates from the ventral tegmental area (VTA) and projects to reward-related regions, including the nucleus accumbens (NAc), subthalamic nucleus (STN), amygdala (AMY), prefrontal cortex (PFC), hippocampus (HIP), and other areas (4–7). Indeed, accumulating neuroimaging evidence has revealed abnormalities in these regions associated to chronic smoking and relapse (8, 9). However, studies that employed diffusion tensor imaging (DTI) to explore the effect of nicotine on the brain microstructure have yielded inconsistent findings. For instance, chronic nicotine consumption has been linked to both decreased fractional anisotropy (FA) (10, 11) and increased FA (12), along with decreased radial diffusivity (RD) (12) and increased RD (13) levels. In addition, a study on nicotine addiction indicated that smokers have decreased FA values in the left anterior and posterior insula cortex-NAc fiber tracts, and higher FA, and lower axial diffusivity (AD), RD, and mean diffusivity (MD) values in the right posterior insula cortex-NAc fiber tracts, when compared to non-smokers (14). Despite these advancements, studies that used neuroimaging for nicotine addiction diagnosis, prognosis and treatment remain scarce. This challenge may be partially attributed to the limited investigations conducted on brain white matter (WM), which constitutes >50% of the total mass of the human brain, and is essential for facilitating connections among various cortical regions (9).

Diffusion magnetic resonance imaging (dMRI) is the unique non-intrusive approach available for evaluating the structural integrity of WM (15). The predominant dMRI technique utilized for studying WM connectivity, and its associations with substance abuse and addictive behaviors is DTI, based on the Gaussian model (12, 16). However, fiber tracking based on DTI has limitations in revealing the crossing or branching fibers in a voxel (17, 18). Diffusion spectrum imaging (DSI) can reliably track the fibers along different directions within a voxel, thereby improving fiber reconstruction, and providing a more accurate reflection of the brain’s complex structures (19). For instance, a study revealed that DSI tractography displayed the lower maturity in the cingulum bundle when myelination was incomplete, while DTI tractography tended to terminate in such regions, possibly due to the presence of crossing fibers (20). Furthermore, DSI provides high-quality fiber tractography, reduces partial volume effects and false continuation artifacts, and has been utilized to reveal more comprehensive connectivity patterns and detailed anatomical structures (21–23). In addition, DSI imaging data can be employed to analyze elementary DTI indicators (24). Therefore, DSI demonstrates a powerful potential for enhancing the tractography of complex WM fibers, and has been increasingly utilized in studying a range of clinical conditions, including psychosis, stroke and epilepsy, among others (25–27). However, reports on the application of DSI in adult nicotine addiction remain rare.

In clinical practice, varenicline, bupropion and nicotine withdrawal management therapy are commonly prescribed for smoking cessation. Although these treatments are effective for a number of smokers, a substantial number of individuals do not respond to these treatments, presenting persistent challenges in addressing relapse (28). To date, alternative treatments with greater efficacy, such as neurostimulation techniques, including transcranial magnetic stimulation (TMS), have been developed for smoking discontinuation (29). Repetitive transcranial magnetic stimulation (rTMS) has been described as a non-invasive, medication-free therapeutic approach rooted in neural circuit modulation (30–32). Although TMS can only stimulate the cortical surface beneath the device’s magnetic coil (33), empirical evidence suggests that TMS can additionally influence the activity in the “downstream” brain regions (34). Furthermore, a recent study that combined functional magnetic resonance imaging (fMRI) and rTMS suggested that the dorsomedial PFC-extended AMY/basal forebrain circuit may be associated to nicotine addiction in schizophrenia and non-schizophrenia individuals (35). Other studies have also suggested the potential effect of rTMS on smoke cessation (36–38). However, the mechanism of rTMS in the treatment of smokers without psychosis has not been well-investigated.

Thus, DSI tractography was utilized to investigate the WM microstructure changes in the mesolimbic dopamine pathway-related regions (VTA, NAc, STN, AMY, PFC and HIP) in smokers who received rTMS treatment. Furthermore, it was hypothesized that rTMS can be beneficial for behavioral improvement, and modulate the WM microstructure in mesolimbic dopamine pathway-related regions. Moreover, a correlation may exist between changes in nicotine addiction behavior and alterations in WM microstructure.

## 2 Methods

### 2.1 Participants

**Figure 1:**
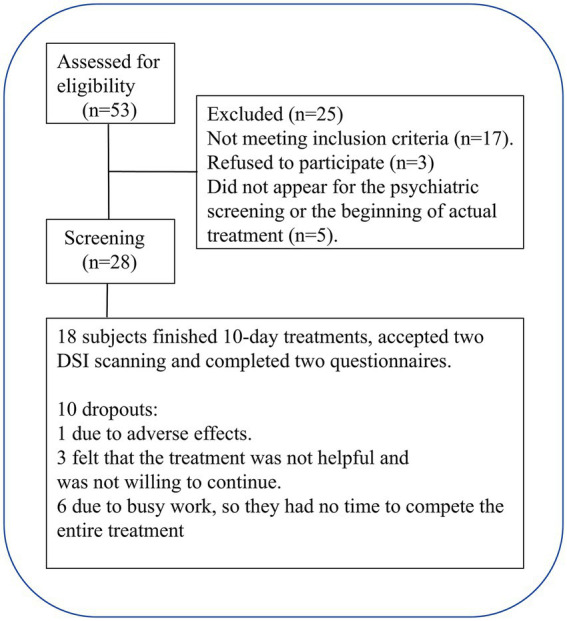
Flowchart for the recruitment of participants.

Fifty-three smokers were engaged via online postings and referrals through verbal communication. Initial screening was conducted through a brief telephone interview, followed by a detailed interview, according to the criteria for inclusion and exclusion (Table 1), and 20 smokers were excluded. Participants who met the inclusion criteria underwent further face-to-face screening, and five smokers failed to attend the psychiatric screening or the initial phase of the actual treatment. Finally, 18 participants finished all the assigned tasks and 10 rTMS treatments. The flowchart for participant recruitment and treatment is presented in Figure 1. The present study was approved by the Institutional Review Board of Zhejiang Hospital (2019 Pro-examination-34K No. -X2). All participants provided a signed consent form.

**Table 1:** Inclusion and exclusion criteria for the study.

Inclusion criteria	Exclusion criteria
Between the age of 18 and 60 years old	Current substance use of any psychoactive substances other than nicotine
Smoking ≥5 cigarettes per day	Contraindications to MRI and TMS
Smoking for >5 years	Used other forms of nicotine delivery, such as nicotine patches and electronic cigarettes
Motivated to quit smoking	Currently taking drugs or smoking cessation medications, including varenicline and bupropion
	History of epilepsy, psychiatric disorders, heart disease, or relevant medical history of other cerebrovascular disorders

MRI, magnetic resonance imaging; TMS, transcranial magnetic stimulation.

### 2.2 Assessment of cigarette consumption, nicotine dependence, and craving levels

Daily cigarette consumption was self-reported by the participants, and nicotine dependence was assessed using the Fagerström Test for Nicotine Dependence (FTND) (39) and smoking severity index (SI) (40). Symptoms related to nicotine withdrawal were evaluated using the Minnesota Nicotine Withdrawal Scale (MNWS) formulated in 1986 (41). Cravings were assessed using the short version of the Tobacco Craving Questionnaire (sTCQ) (42) and a subjective visual analogue scale (VAS) (43, 44). The FTND, SI, MNWS and sTCQ were measured twice: at baseline before rTMS (S1) and after the 10th rTMS session (S2). VAS was assessed before and after each rTMS session through the responses of the participants to the following question: “How much do you want to smoke right now?”

### 2.3 The rTMS procedure

The rTMS treatment was conducted according to prior studies (31, 45) by targeting the left dorsolateral prefrontal cortex (DLPFC) using a YRD CCY-II TMS (Yiruide, China) stimulator equipped with an O-coil design. The resting motor threshold (RMT) was established through motor strip activation, followed by the analysis of the resultant motor-evoked potential recorded from the right abductor pollicis brevis (APB). The stimulation site was identified using a TMS head cap. The stimulation lasted for 800 s, and delivered 1,800 pulses that comprised 36 trains of 50 pulses at 10 Hz, with an inter-train interval of 20 s, through an intensity set at 100% of the RMT. Each participant received 10 treatments over 2 weeks.

### 2.4 Imaging acquisition and processing

The magnetic resonance imaging (MRI) scan was conducted using a 3 T MRI scanner (Magnetom Skyra, Siemens, Germany) equipped with a 32-channel head coil. During the MRI, the participants were instructed to remain motionless, close their eyes, and avoid focusing on any specific thought. Motion artifacts were minimized by placing a foam pad between the head and coil. Three-dimensional (3D) T1-weighted images were acquired using the following settings: 176 sagittal slices, gap = 0 mm, field of view (FOV) = 256 × 256 mm, repetition time (TR) = 1,900 ms, echo time (TE) = 2.50 ms, inversion time (TI) = 900 ms, flip angle = 30°, and voxel size = 1.00 × 1.00 × 1.00 mm^3^. DSI scans were acquired using the following parameters: slice number = 60, TR = 8,700 ms, TE = 110.00 ms, FOV = 240 × 240 mm, voxel size = 1.10 × 1.10 × 2.50 mm^3^, phase encoding direction of anterior-to-posterior (A to P), maximum *b*-value = 3,000 s/mm^2^, q-space diffusion mode, q-space weightings = 3, and half q-space coverage.

The dMRI data was preprocessed by eddy current and motion correction using DSI Studio.^1^ Then, the diffusion data underwent reconstruction, utilizing the generalized q-sampling imaging (46), according to a previous study (12). The quantitative anisotropy (QA), FA, MD, AD, and RD values of each participant were computed using DSI Studio.

### 2.5 Regions of interest-based fiber tracking

**Figure 2:**
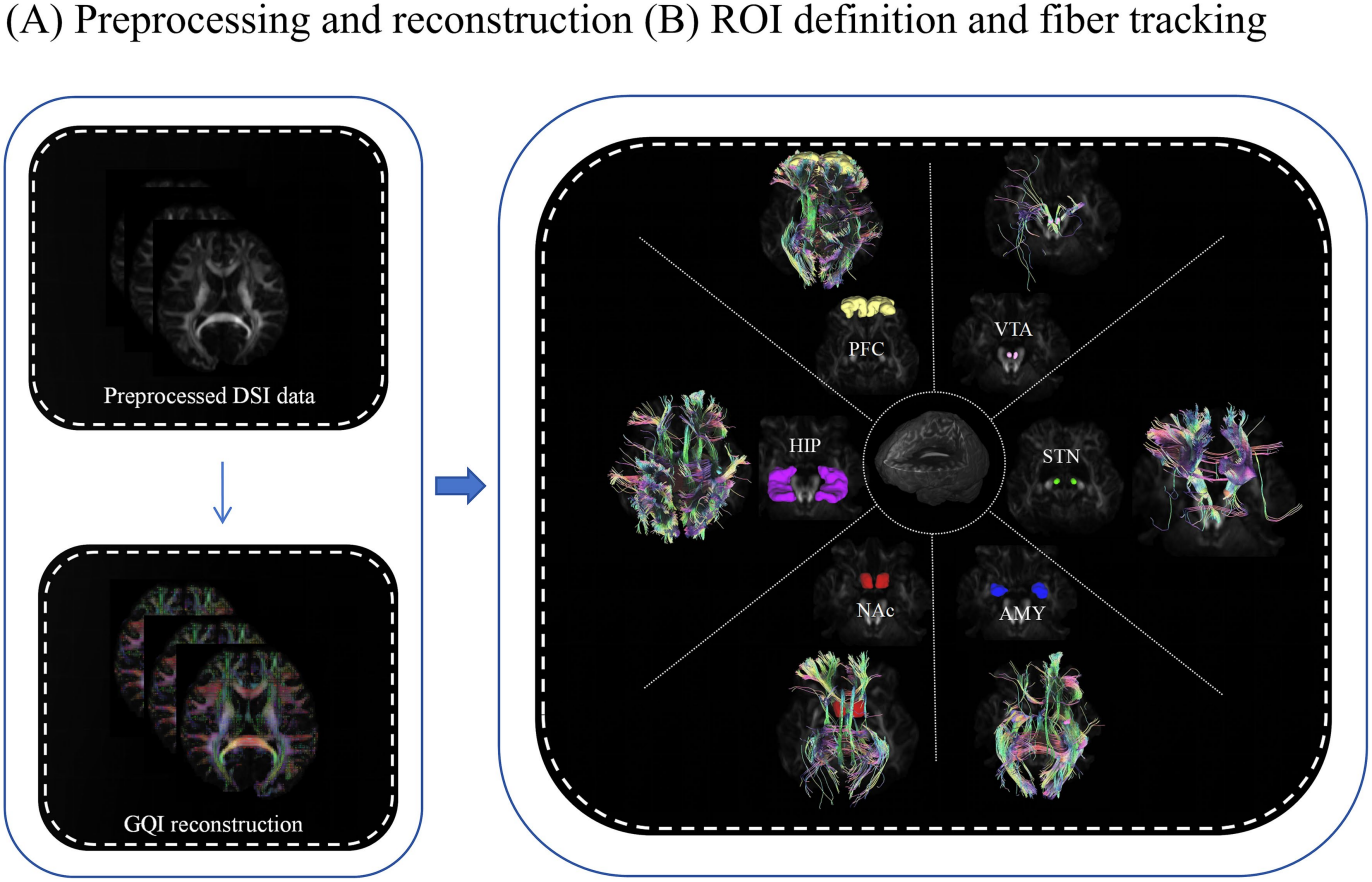
Working flowchart for image processing. **(A)** Preprocessing and reconstruction. **(B)** Definition of ROIs and tracking fibers using the DSI studio software. The above processes were prepared for collecting the diffusion measures for statistical analysis. ROI, region of interest; GQI, generalized q-sampling imaging; PFC, prefrontal cortex (yellow); HIP, hippocampus (purple); NAc, nucleus accumbens (red); AMY, amygdala (blue); STN, subthalamic nucleus (green); VTA, ventral tegmental area (pink).

The fiber clusters of six regions of interest (ROIs) related to nicotine addiction circuits (VTA, NAc, STN, AMY, PFC and HIP) were chosen from the Anatomical Automatic Labeling (AAL) template. Linear and non-linear transformations were applied to map the QA, FA, MD, AD and RD image coordinates of each participant’s brain onto the Montreal Neurological Institute’s (MNI) space. The working flowchart is presented in Figures 2A,B.

### 2.6 Statistical analysis

The data was expressed in mean ± standard error of the mean (SEM), and analyzed using the Statistical Package for the Social Sciences (SPSS, version 31; IBM, Armonk, NY, United States). Paired *t*-test was conducted to assess continuous variables (outcomes), which included the FTND, SI, MNWS, sTCQ and VAS scores, and the diffusion metrics along the fiber tracts of ROIs between S1 and S2. Network-based statistics (NBS) (47) correction was adopted for the ROIs, according to the following steps: forming a set of supra-threshold edges using an initial threshold (*p <* 0.001) in the paired *t*-test matrix, and determining the statistical significance of each component’s size using non-parametric permutation testing (1,000 permutations), which provides strong control over the family-wise error rate (FWER). ROIs that exhibited significant changes in the diffusion metrics (QA, FA, MD, AD and RD) following the rTMS treatment were further analyzed for Pearson correlation with various tobacco addiction behavioral scales. Then, a multiple linear regression model was used to assess the association between the changes in these diffusion metrics and changes in tobacco addiction behavioral scales, controlling for the potential confounding effects of age. A *p*-value of <0.05 was set as the level of significance.

## 3 Results

### 3.1 Demographic characteristics

The present study included a total of 18 male smokers with an age of 39.56 ± 2.71 years old, education duration of 11.83 ± 0.85 years, and smoking age of 19.17 ± 2.28 years old. The ethnic groups of the participants included the Han population (*n* = 17) and other populations (*n* = 1).

### 3.2 Treatment effects of rTMS on nicotine dependence and craving

**Figure 3:**
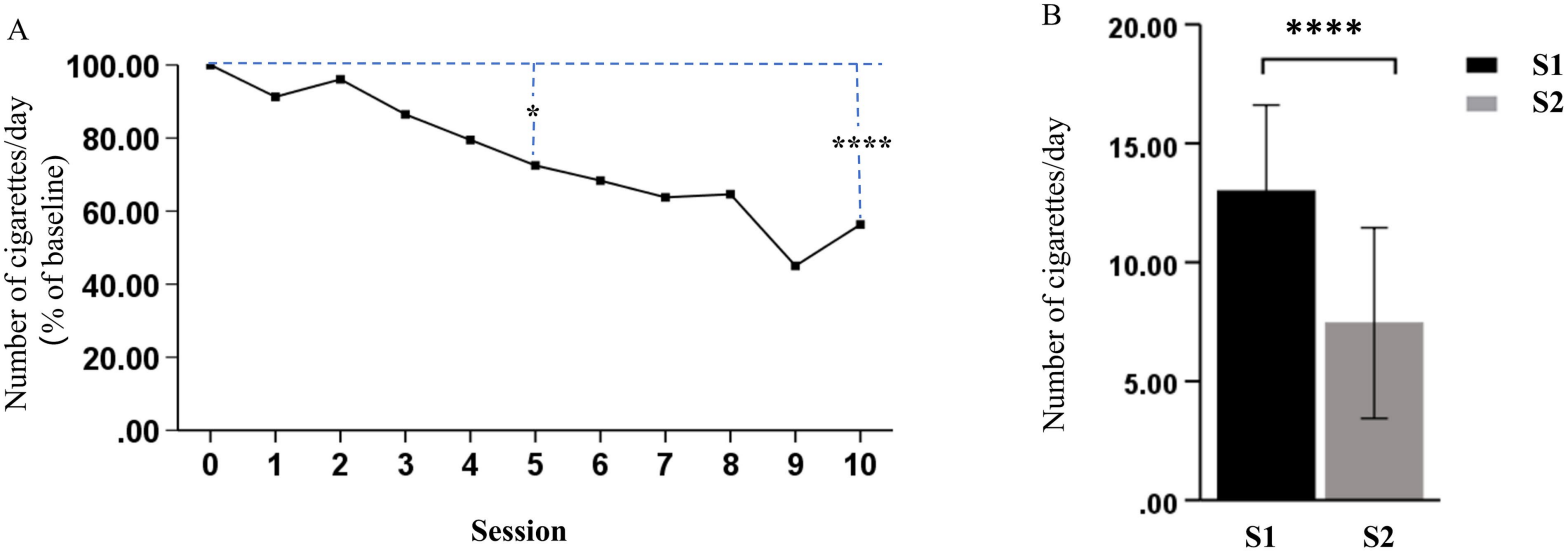
Effects of rTMS on cigarette consumption. **(A)** Daily change (% of baseline) in the number of cigarettes smoked for each treatment session. **(B)** The number of cigarettes smoked per day significantly decreased after the last treatment session (S2), when compared to before the first treatment session (S1) (^*^p < 0.05 and ^****^p < 0.0001).

As shown in Table 2, the rTMS (S2) significantly decreased nicotine dependence at S2, as demonstrated by the significant decrease in FTND (*p* < 0.0001) and SI (*p* < 0.0001), when compared to that at S1. The MNWS scores indicated that the syndrome related to nicotine withdrawal significantly decreased between S1 and S2 (*p* = 0.024). In addition, the sTCQ (*p* < 0.0001) and VAS (*p* < 0.0001) scores indicated that the nicotine cravings significantly decreased after rTMS, when compared to S1. These results suggest that rTMS was potentially effective in smoking cessation, as supported by the self-reported cigarette consumption (Figure 3). Figure 3A shows that there was a decreasing trend of daily cigarette consumption following rTMS treatments, when compared to baseline (S1), and this trend reached a significantly different level after session 5. Figure 3B shows that there was a significant difference in cigarette consumption between S1 and S2. Among the 18 participants, four participants completely stopped smoking after 2 or 3 days during the study period, and all other participants presented with gradually decreased cigarette consumption.

**Table 2:** Effects of rTMS in smokers.

Clinical scales	S1 (*n* = 18)	S2 (*n* = 18)	*p*-value Hedges’*g* 95% CI LL 95% CI UL
FTND	3.67 ± 0.49	1.67 ± 0.34	<0.0001 1.14 0.55 1.71
SI	2.67 ± 0.39	1.11 ± 0.27	<0.0001 1.11 0.53 1.68
MNWS	9.28 ± 1.66	5.72 ± 1.17	0.0240 0.56 0.07 1.03
sTCQ	51.33 ± 3.50	33.00 ± 3.48	<0.0001 1.24 0.63 1.84
VAS	48.06 ± 5.67	12.67 ± 3.32	<0.0001 1.40 0.70 1.96

The data are presented in mean ± standard error of the mean (SEM). CI, confidence interval; LL, lower limit; UL, upper limit; rTMS, repetitive transcranial magnetic stimulation; S1, stage before rTMS treatment; S2, stage after the 10th rTMS treatment; FTND, the Fagerström Test for Nicotine Dependence; SI, smoking severity index (SI); MNWS, the Minnesota Nicotine Withdrawal Scale; sTCQ, the short version of the Tobacco Craving Questionnaire; VAS, visual analogue scale.

### 3.3 Effects of rTMS on the dMRI metrics of smokers

The analysis of the image metrics of MRI of smokers before and after rTMS treatment revealed a significant decrease in AD values in the right NAc fiber tracts after rTMS treatment (*p* = 0.03, Hedges’*g* = 0.55, Table 3). However, the other image metrics, including QA, FA, MD and RD, did not significantly change after rTMS in the right NAc fiber tracts. In addition, the other nicotine addiction-related ROIs, including VTA, STN, AMY, PFC and HIP, were not significantly affected by the rTMS treatment, when compared to those between S1 and S2 (all *p* > 0.05, Hedges’*g* < 0.5, Table 3).

**Table 3:** Paired *t*-test results of dMRI metrics in smokers before and after rTMS (after correction).

ROI		QA	FA	MD	AD	RD
L-VTA	*p*	0.82	0.94	0.79	0.82	0.77
	Hedges’*g*	0.06	0.02	0.07	0.05	0.07
	95% CI (LL, UL)	−0.39, 0.49	−0.46, 0.42	−0.50, 0.38	−0.49, 0.39	−0.51, 0.38
R-VTA	*p*	0.88	0.97	0.15	0.09	0.21
	Hedges’*g*	0.04	0.01	0.36	0.42	0.31
	95% CI (LL, UL)	−0.41, 0.48	−0.45, 0.43	−0.80, 0.12	−0.86, 0.06	−0.75, 0.16
L-STN	*p*	0.64	0.94	0.89	0.79	0.95
	Hedges’*g*	0.11	0.02	0.03	0.07	0.01
	95% CI (LL, UL)	−0.34, 0.55	−0.46, 0.42	−0.41, 0.47	−0.38, 0.50	−0.43, 0.45
R-STN	*p*	0.80	0.17	0.85	0.47	0.93
	Hedges’*g*	0.06	0.33	0.04	0.17	0.02
	95% CI (LL, UL)	−0.38, 0.50	−0.77, 0.14	−0.48, 0.40	−0.61, 0.28	−0.42, 0.46
L-NAc	*p*	0.46	0.33	0.70	0.80	0.64
	Hedges’*g*	0.18	0.24	0.09	0.06	0.11
	95% CI (LL, UL)	−0.61, 0.28	−0.67, 0.22	−0.35, 0.53	−0.39, 0.50	−0.34, 0.55
R-NAc	*p*	0.53	0.38	0.09	0.03*	0.16
	Hedges’*g*	0.15	0.21	0.43	0.55	0.35
	95% CI (LL, UL)	−0.30, 0.59	−0.25, 0.65	−0.06, 0.87	0.05, 1.00	−0.13, 0.78
L-AMY	*p*	0.75	0.85	0.35	0.22	0.44
	Hedges’*g*	0.08	0.04	0.23	0.30	0.19
	95% CI (LL, UL)	−0.51, 0.37	−0.48, 0.40	−0.66, 0.23	−0.73, 0.17	−0.62, 0.27
R-AMY	*p*	0.73	0.46	0.53	0.71	0.45
	Hedges’*g*	0.08	0.18	0.15	0.09	0.18
	95% CI (LL, UL)	−0.37, 0.52	−0.28, 0.61	−0.59, 0.30	−0.53, 0.36	−0.62, 0.27
L-HIP	*p*	0.97	0.30	0.88	0.96	0.79
	Hedges’*g*	0.05	0.24	0.11	0.07	0.13
	95% CI (LL, UL)	−0.49, 0.40	−0.67, 0.23	−0.34, 0.55	−0.37, 0.51	−0.32, 0.57
R-HIP	*p*	0.94	0.27	0.31	0.40	0.28
	Hedges’*g*	0.06	0.15	0.30	0.28	0.30
	95% CI (LL, UL)	−0.49, 0.39	−0.59, 0.30	−0.17, 0.73	−0.19, 0.71	−0.17, 0.73
L-PFC	*p*	0.96	0.47	0.58	0.23	0.91
	Hedges’*g*	0.01	0.17	0.13	0.29	0.03
	95% CI (LL, UL)	−0.43, 0.45	−0.28, 0.61	−0.32, 0.57	−0.18, 0.73	−0.42, 0.47
R-PFC	*p*	0.75	0.23	0.78	0.40	0.54
	Hedges’*g*	0.08	0.29	0.07	0.20	0.15
	95% CI (LL, UL)	−0.37, 0.51	−0.18, 0.73	−0.51, 0.38	−0.25, 0.64	−0.58, 0.30

^*^p < 0.05; L, left; R, right; CI, confidence interval; LL, lower limit; UL, upper limit; ROI, region of interest; QA, quantitative anisotropy; FA, fractional anisotropy; MD, mean diffusivity; AD, axial diffusivity; RD, radial diffusivity; VTA, ventral tegmental area; STN, subthalamic nucleus; NAc, nucleus accumbens; AMY, amygdala; HIP, hippocampus; PFC, prefrontal cortex.

### 3.4 Correlation and regression analysis of treatment outcomes

**Figure 4:**
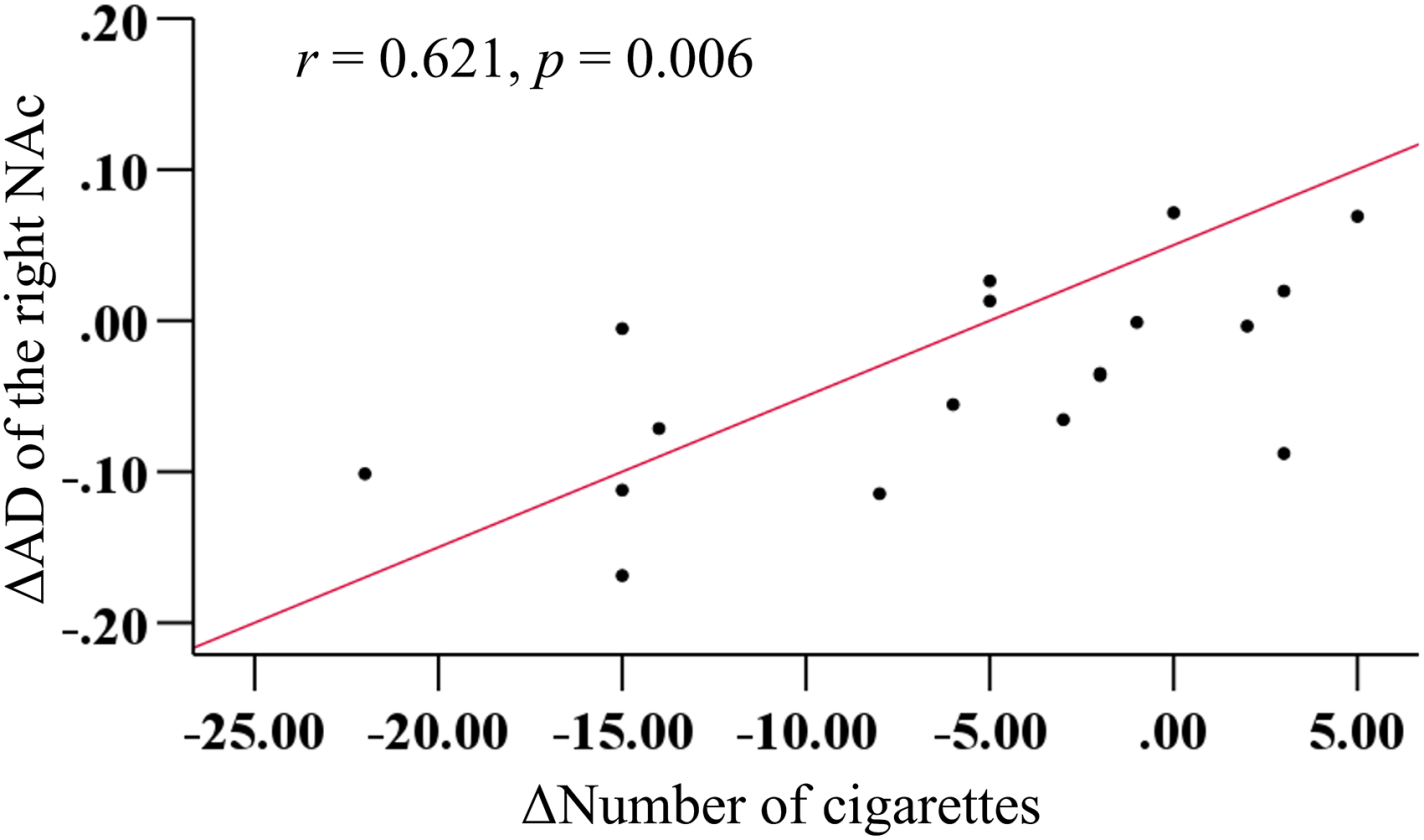
More decrease in cigarettes consumption predicted more reduction in AD in the right NAc fibers after rTMS treatment.

There was no significant correlation between AD values in the right NAc fibers and various tobacco addiction behavioral scales, either at pre- or post-treatment (all *p* > 0.05). However, a strong positive correlation (*r* = 0.621, *p* = 0.006) was observed between the change in cigarette consumption and change in AD values in the right NAc fibers before and after rTMS treatment (Figure 4). Furthermore, the multiple linear regression yielded a standardized model, in which the change in AD values of the right NAc fibers was primarily predicted by the change in cigarette consumption (*β* = 0.608, *p* = 0.009), with age making a minimal contribution (*β* = −0.089, *p* = 0.666): Z_ΔAD = (0.608 × Z_Δcigarette) + (−0.089 × Z_age).

### 3.5 Power analysis

A *post-hoc* power analysis using the AD values of the right NAc fibers before and after TMS treatment in smokers was conducted, with the *α* error probability set at 0.05. The achieved statistical power (1 − *β*) was calculated via the G*Power software, and the result was 0.6. This result indicates that the statistical power of the present study only reached a moderate level. Subsequently, *a priori* power analysis was performed using the same effect size (*d* = 0.554, derived from the pre- and post-treatment AD values of the right NAc fibers), with the *α* error probability set to 0.05, and the desired power (1 − *β*) set to 0.8. The analysis indicated that a minimum sample size of 28 participants would be required to achieve adequate power in future studies.

## 4 Discussion

Smoking and nicotine addiction are worldwide problems (1–3). Recent studies have indicated that nicotine addiction can lead to abnormalities in the brain, including its structures and functions (9, 48), as detected by advanced imaging techniques, such as fMRI, DTI and DSI (11, 12, 24, 35). However, there is no satisfactory strategy for smoking cessation. rTMS is a novel non-invasive technique, which has been used in multiple diseases (24–26). The present study delved in determining whether rTMS can influence the WM microstructure in mesolimbic dopamine pathway-related regions in individuals with nicotine dependence. The findings revealed that cigarette consumption and the questionnaire scores decreased after rTMS treatment, suggesting that rTMS treatment is a potential effective intervention for reducing nicotine dependence and craving in smokers, and can partially alleviate the anxiety and depression induced by nicotine withdrawal. Furthermore, the present study revealed that AD decreased in the right NAc fiber tracts after short-term rTMS treatment, suggesting that rTMS may have the potential to change the WM microstructure, and thereby decrease nicotine addiction, although there were no significant changes in QA, FA, MD and RD in NAc and other dopamine pathway related regions.

Previous studies have indicated that chronic nicotine exposure diminishes communication throughout the brain, and increases local connectivity between specific network nodes (49). The NAc receives direct glutamatergic projections from the AMY, HIP, thalamus and PFC, and indirect mesolimbic dopaminergic projections from the VTA and substantia nigra (50). Furthermore, the NAc plays a critical role in neural mechanisms of addiction, primarily due to dopamine release, and the activation of multiple receptors within the NAc (50). Emerging evidence suggests that structural and functional changes can occur in the NAc of subjects with substance use disorders. For example, previous studies have reported the decrease in structural volume of the left NAc (51–53), and the increase in thickness of the right VTA-NAc (53) in subjects with heroin addiction. Similarly, the present study indicated that rTMS significantly decreased AD in the right NAc fiber tracts in smokers with lower cigarette consumption, suggesting that the right NAc is involved in nicotine addiction.

It has generally been considered that AD primarily reflects axonal integrity, with decreased AD indicating a reduction in axonal volume, and the beginning stages of axonal damage (54, 55). However, the increase in volume of glial cells may also be correlated to lower AD (56), and nicotine exposure may increase the number and density of glial cells (57). In addition, a decrease in AD may indicate early neural immune activation or the initial phases of intracellular inflammation and edema (55). Therefore, this phenomenon was attributed to the fact that the majority of participants (14 of 18 participants) continued smoking throughout the treatment period, despite the reduction in the number of cigarettes smoked, and merely four participants ceased smoking completely in the last 2 or 3 days of the study period. A previous study suggested that long-term smoking can compromise the integrity of WM in the brain (58). However, there is no definitive consensus on the exact duration or number of days required for this effect to manifest (49, 59). The present study indirectly demonstrated that nicotine intake over a period of approximately 2 weeks may initially influence the WM microstructure of the right NAc fibers. However, several studies have reported that the WM of the left hemisphere may be more vulnerable to the neurotoxic effects of nicotine, when compared to that of the right hemisphere (14, 58). These results were inconsistent with the present results, in which the right NAc fibers may be more vulnerable to nicotine. In addition, the Pearson correlation analysis indicated that the greater the reduction in cigarette consumption, the greater the decrease in AD values in the right NAc fibers after rTMS treatment. Nonetheless, the results may not be fully aligned with the previous explanation of the investigators. If the reduction in AD values was attributed entirely to the fact that most subjects continued to smoke during the treatment period, the more the cigarette consumption decreased, the less the AD values should have decreased. Therefore, the investigators proposed a hypothesis that rTMS may alter the microstructure of the right NAc fibers and modulate dopamine release within the NAc, thereby reducing nicotine dependence and craving in smokers. A study reported that the reduction in dopamine release in the NAc decreased the nicotine-taking behavior (60). In contrast, a decrease in AD does not necessarily indicate the presence of axonal injury, and reductions in AD have been observed in healthy brain maturation (9). Meanwhile, the age-related reduction of AD in a number of brain regions have been reported, which may be correlated to the growth of neurofibrils, such as microtubules and neurofilaments (61, 62). However, the results of the multiple linear regression analysis indicated that age had a minimal contribution to the reduction of AD in the right NAc fibers in the present study. This aligns with the perspective that axon coherence, which is associated to AD, does not serve as a major contributing factor to the developmental changes observed in numerous studies (63). Hence, the significant reduction in AD values in the right NAc fiber tracts in smokers after rTMS treatment may indicate that rTMS may facilitate the growth of neurofibrils, and modulate the microstructure of the right NAc fiber tracts, excluding the influence of age. The previous study conducted by the investigators on functional MRI revealed that rTMS can increase the functional connectivity of the right NAc with several visual and sensory processing regions in smokers, and that the right NAc appeared to play a central role in the adaptive reorganization of brain networks during the cessation of smoking through rTMS treatment (64). Therefore, the changes in AD are likely to reflect the complex interactions among multiple biological factors that influence its progression in various directions (56). The present study may provide crucial new insights into the neural mechanisms of rTMS in treating smoking addiction. In future studies, the role of NAc WM laterality, particularly in its relation to smoking and rTMS, warrants further investigation.

For the right insula-NAc fiber, a study reported lower FA values in individuals with heroin addiction, when compared to healthy controls (65). Furthermore, DTI studies have revealed reduced FA in tracts projecting from the bilateral NAc to the frontal cortex in individuals with nicotine addiction, when compared to healthy controls (66, 67). Compared to non-smokers, smokers exhibit an increased integration coefficient between the frontoparietal network (FPN) and basal ganglia network (BGN), and a reduced integration coefficient between the medial frontal network (MFN) and FPN. Furthermore, the analysis of variance revealed that rTMS treatment can reduce the integration coefficient between the FPN and BGN, and improve the recruitment coefficient of the FPN (68). These studies indicate that nicotine addiction involves other brain regions. However, the present study did not detect significant changes in QA, FA, MD and RD in the right NAc, and all metrics in other brain regions, which was inconsistent with the expectations of the investigators. This discordance may have been primarily derived from the following reasons: (1) the different indicators exhibited varying sensitivities to different diseases (19); (2) there was a possibility that nicotine did not damage the relevant fibers; (3) the rTMS treatment required more repetitions to demonstrate its effect. These factors may also account for the mismatch between robust behavioral improvements and limited imaging changes. Furthermore, the placebo effect could have contributed to this phenomenon. Therefore, sham stimulation experiments should be conducted (31, 35).

## 5 Limitations

There were some limitations in the present study that require clarification. First, the sample size was small. Thus, a large sample study is required to validate the present findings. Second, there was no control group, such as sham treatment and non-smokers. Third, further research that involves female smokers is necessary to ensure the applicability of the present findings across genders, although it is difficult to recruit female smokers in China. Fourth, the absence of objective biochemical validation limited the precision of the present experimental findings. Incorporating established biochemical markers in future studies, such as CO/cotinine (14, 35), would enhance the accuracy and reliability of the research outcomes. Fifth, the participants should be stratified by age, since a study reported that smokers are generally associated to lower FA in adults, and higher FA in young adults (≤30 years old) (69). Sixth, more repetitions of the rTMS treatment and follow-up may be helpful to verify the present results. Finally, other regions should be carefully investigated for whole-brain tractography, since the NAc receives projections from the medial PFC, anterior insula, AMY and VTA, contributing to its intricate anatomical connections within addiction circuits (50).

## 6 Conclusion

The present study was the first attempt to determine how rTMS affects the brain microstructure in individuals with nicotine dependence using DSI. The results indicated that the decreased AD values within the right NAc fiber tracts could be the “result” of nicotine addiction, or directly attributable to HF-rTMS. These results suggest that the right NAc emerged as a region of interest that warrants further investigation as a potential therapeutic target.

## Data Availability

The raw data supporting the conclusions of this article will be made available by the authors, without undue reservation.

## Footnotes

^1^ http://dsi-studio.labsolver.org
